# Alterations in T lymphocyte subsets and mitochondrial-related parameters in patients with chronic kidney disease

**DOI:** 10.3389/fmed.2026.1811069

**Published:** 2026-06-19

**Authors:** Sizeng Bao, Chunlei Luo, Hanlu Wang, Jianwei Ma, Xueyan Bian

**Affiliations:** Department of Nephrology, The First Affiliated Hospital of Ningbo University, Ningbo, Zhejiang, China

**Keywords:** chronic kidney disease, CKD, mitochondrial mass, mitochondrial membrane potential, T lymphocyte subsets

## Abstract

**Introduction:**

To investigate the association between renal function and T lymphocyte subsets and mitochondrial-related parameters in patients with chronic kidney disease (CKD).

**Methods:**

This retrospective study included patients with CKD between July 2023 and June 2024. Disease stage was assessed based on estimated glomerular filtration rate (eGFR), and patients were classified into CKD stages 1–5 accordingly. Peripheral blood samples were analyzed by flow cytometry to assess T lymphocyte subsets and mitochondrial-related parameters, including mitochondrial mass (MM) and the percentage of cells with low mitochondrial membrane potential (MMP-Low%).

**Results:**

A total of 802 patients with CKD were included (440 males; mean age 56.3 ± 16.0 years), comprising 226 stage 1, 204 stage 2, 205 stage 3, 82 stage 4, and 85 stage 5 cases. Lymphocyte percentage was lower and neutrophil percentage higher in stage 5 vs. stages 1–2 (both *p* < 0.001). Absolute counts of CD45^+^, CD3^+^, CD4^+^, and CD8^+^ T cells were significantly lower in stages 3–5 vs. 1–2 (all *p* < 0.05). Mitochondrial alterations in stages 3–5 included decreased MMP-Low% across CD3^+^, CD4^+^, and CD8^+^ subsets (all *p* < 0.05). After multivariable adjustment, eGFR was independently associated with CD3^+^ T-cell MMP-Low% (β = 0.0375, *p* = 0.024) and CD8^+^ T-cell MMP-Low% (β = 0.0826, *p* < 0.001), whereas the association with CD8^+^ T-cell MM was no longer statistically significant.

**Conclusion:**

Reduced T lymphocyte counts and alterations in mitochondrial-related parameters, particularly membrane potential-related parameters, were observed in patients with CKD and were associated with more advanced CKD stages.

## Introduction

Chronic kidney disease (CKD) is a major global public health problem, with global prevalence and mortality increasing steadily over the past three decades (1, 2). Global burden of disease (GBD) 2021 data show that CKD prevalence and mortality have risen by approximately 40% from 1990 to 2021, reflecting a rapidly growing global disease burden (3). CKD often progresses from early asymptomatic stages to more advanced stages due to its insidious onset, ultimately leading to end-stage renal disease (ESRD) (4, 5). By 2021, the global disability-adjusted life-years (DALYs) attributed to CKD had nearly doubled compared with 1990, further highlighting its escalating burden (3). Beyond epidemiological measures, chronic kidney disease is clinically characterized by progressive loss of renal function and is associated with substantially increased risks of adverse cardiovascular outcomes and premature mortality, with both early and advanced CKD independently conferring elevated risk of major cardiovascular events, including sudden cardiac death (6–8).

The primary pathological features of CKD include progressive inflammation, oxidative stress, and renal fibrosis (1, 9). Renal fibrosis is a common pathological pathway of CKD progression and involves maladaptive tissue repair, excessive extracellular matrix deposition, immune-cell infiltration, and activation of profibrotic signaling pathways (9–12). Oxidative stress, inflammatory signaling, and metabolic or gut microbiota-related mechanisms may further contribute to renal inflammation and fibrotic remodeling (13–17). Consequently, CKD progression is closely linked to significant alterations in the immune system (18). Studies on immune cell changes in advanced CKD have shown a significant decrease in lymphocytes, including CD4^+^ and CD8^+^ T-lymphocyte subsets (19), a reduction in B cell proportions, and an increase in NK cell proportions (20). Advanced CKD is associated with decreased thymic output and increased apoptosis, leading to a reduction in naive T lymphocytes and an increase in effector memory T lymphocytes, which contributes to heightened inflammation (21). Mitochondrial function plays a central role in regulating T-cell activation, differentiation, and metabolic programming, and mitochondrial dysfunction has been increasingly recognized as a key contributor to immune dysregulation and chronic inflammatory states (22).

However, few studies have comprehensively evaluated the relationship between mitochondrial function-related changes in peripheral blood immune cells and CKD progression. Mitochondria are dynamic organelles crucial for adenosine triphosphate (ATP) synthesis, metabolic regulation, production of reactive oxygen species (ROS), and cell differentiation and death (23). Mitochondrial mass (MM) and percentage of cells with low mitochondrial membrane potential (MMP-Low%) are commonly used flow cytometry-based indicators related to mitochondrial function. Within physiological conditions, MM and MMP-Low% are commonly used as indicators of mitochondrial status; however, their interpretation is context-dependent, and alterations observed under chronic inflammatory or metabolic stress may reflect dysfunctional or compensatory mitochondrial remodeling rather than enhanced bioenergetic activity (24, 25).

Flow-cytometric mitochondrial dyes have been widely used to assess mitochondrial content and membrane potential in immune cells (26–29). MM and MMP-Low% of peripheral T lymphocytes have been applied in studies of sepsis, severe SARS-CoV-2 infection, chronic hepatitis B virus infection, HIV, and dermatomyositis (30–34). Recent clinical studies have reported CKD-related alterations in T lymphocyte subsets and related mitochondrial indicators. However, current evidence does not adequately address how these immunometabolic changes vary across CKD stages or relate to renal impairment (19, 35). This gap limits understanding of CKD-related immune dysfunction. Therefore, this study aimed to evaluate T lymphocyte subsets and mitochondrial-related parameters across CKD stages and examine their associations with renal function.

## Materials and methods

### Study subjects

This retrospective study included patients with CKD who underwent flow cytometry-based assessment of T lymphocyte mitochondrial-related parameters at the First Affiliated Hospital of Ningbo University between July 2023 and June 2024. Inclusion criteria were: 1. age ≥ 18 years; 2. diagnosed with CKD according to Kidney disease improving global outcomes (KDIGO) 2012 Clinical Practice Guidelines; 3. availability of complete relevant test data including flow cytometry-based T lymphocyte mitochondrial-related assessment and renal function test. Exclusion criteria were: 1. presence of acute symptomatic infection, active immune disease, or use of immunosuppressants; 2. undergoing dialysis or kidney transplant recipients. Data from flow cytometry-based T lymphocyte mitochondrial-related assessments were routinely collected as part of the clinical workup for patients with CKD at our center and were available in the electronic medical records.

The estimated glomerular filtration rate (eGFR) was calculated using the Chronic Kidney Disease Epidemiology Collaboration (CKD-EPI) formula based on serum creatinine. CKD stages 1–5 were defined according to KDIGO 2012 Clinical Practice Guidelines (36). Patients were categorized into three groups: early CKD (stages 1–2), middle CKD (stages 3–4), and ESRD (stages 5). Grouping CKD stages into stages 1–2, stages 3–4, and stage 5 was performed to enhance statistical power, minimize variability due to small subgroup sizes, and align the analysis with clinically relevant transitions in renal function.

This study was approved by the Ethics Committee of the First Affiliated Hospital of Ningbo University (2025-205RS) and conducted in accordance with the Declaration of Helsinki. Patient confidentiality was maintained by anonymizing the data. Due to the retrospective nature of the study and the absence of patient interventions, the study was approved for exemption from informed consent by the Ethics Committee of the First Affiliated Hospital of Ningbo University.

### Data collection

Medical records were reviewed to record patient demographics and clinical data, including gender, age, height, weight, underlying diseases, high-sensitivity C-reactive protein, white blood cell count, neutrophil percentage, lymphocyte percentage, hemoglobin, platelet count, serum albumin, globulin, creatinine, and uric acid. Body mass index (BMI) and eGFR were calculated.

Mitochondrial-related data for T lymphocytes in peripheral blood were collected using flow cytometry as reported by others (30–32, 37). The analyzed lymphocyte variables included the percentage and absolute counts of CD3^+^, CD4^+^, CD8^+^, CD4^+^PD-1^+^, and CD8^+^PD-1^+^ T lymphocytes. Mitochondrial-related parameters were evaluated using MM and mitochondrial membrane potential, expressed as MMP-Low%. These flow cytometry–based indicators reflect mitochondrial content and membrane potential distribution, respectively, and serve as indirect surrogate markers of mitochondrial status rather than direct measures of mitochondrial respiratory function, ATP production, reactive oxygen species generation, or mitochondrial dynamics.

Peripheral blood samples were collected in Ethylenediaminetetraacetic acid (EDTA)-anticoagulated tubes and analyzed using a commercially available reagent kit compatible with the NovoCyte DiagCyto 6C2L flow cytometry system (UBBIO Technology, Hangzhou, China). Lymphocytes were gated based on forward and side scatter characteristics in combination with CD45 expression. From the lymphocyte gate, T lymphocytes were identified by CD3 expression and further classified into CD4^+^ and CD8^+^ subsets. CD279 (PD-1) expression was used to further stratify CD4^+^ and CD8^+^ T cells into PD-1–positive populations.

Absolute lymphocyte counts were obtained using the instrument's built-in volumetric counting method. MM was quantified using the median fluorescence intensity of mitochondrial staining within the total gated population, while MMP-Low% was defined as the proportion of cells within the lower mitochondrial membrane potential population based on fluorescence intensity thresholds, consistent with previously established methodologies (31, 33, 38–40).

### Statistical analysis

Statistical analyses were performed using R software, version 4.1.3 (R Foundation for Statistical Computing, Vienna, Austria), (http://www.R-project.org) and GraphPad Prism 10.0 software GraphPad Prism 10.0 software (GraphPad Software, Boston, MA, USA). Variables are expressed as mean ± standard deviation for continuous variables with a normal distribution, M (Q1, Q3) for those with a non-normal distribution, or percentage for categorical variables. Group comparisons were conducted using one-way analysis of variance (ANOVA), Kruskal-Wallis *H*-test or Chi-square tests. The Tukey's *post hoc* critical difference test was used to identify the significant differences between groups in the case of significant *F* values for ANOVA. Spearman correlation analysis and multiple linear regression analysis were used to examine the relationships between immune cell counts, mitochondrial-related parameters, and eGFR. To account for multiple comparisons, *p*-values from correlation analyses were adjusted using the Benjamini-Hochberg false discovery rate (FDR) method and Bonferroni correction. For pairwise comparisons across CKD stages, Holm-Bonferroni correction was additionally applied as a sensitivity analysis. For linear regression models, model diagnostics were performed to assess multicollinearity, residual distribution, and robustness. Variance inflation factors (VIFs) were used to evaluate collinearity, residual distribution was assessed using residual diagnostic plots and Q-Q plots, and robustness was examined using heteroscedasticity-consistent standard errors. The restricted cubic splines were performed to explore the nonlinearity using R. All statistical tests were bilateral, and a *p*-value < 0.05 was considered statistically significant.

## Results

### Patient selection and baseline characteristics

**Figure 1:**
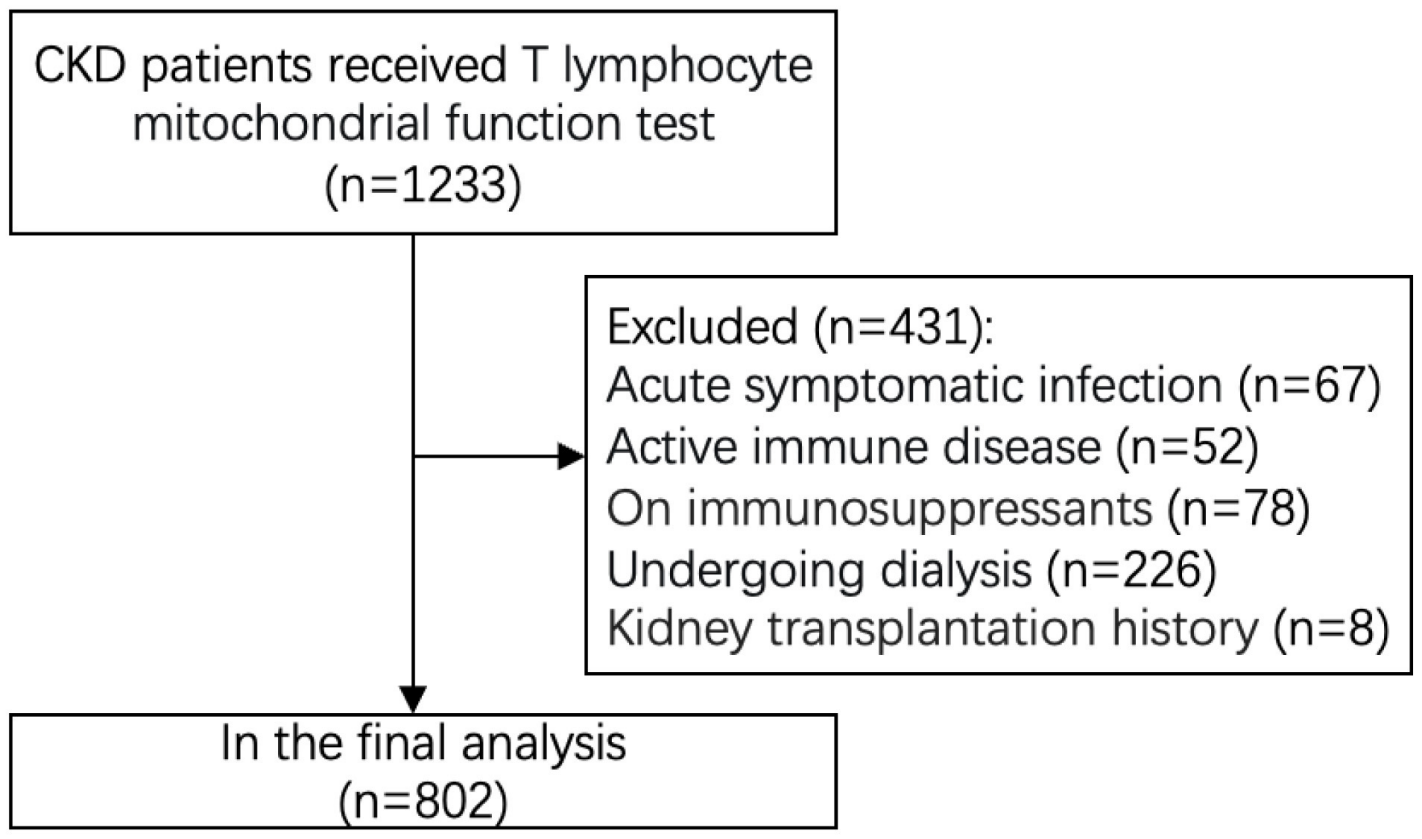
Flowchart of patient selection. A total of 1,233 patients with CKD who underwent T lymphocyte mitochondrial-related parameter testing were screened. After excluding patients with acute symptomatic infection (*n* = 67), active immune disease (*n* = 52), use of immunosuppressants (*n* = 78), ongoing dialysis (*n* = 226), or a history of kidney transplantation (*n* = 8), 802 patients were included in the final analysis.

A total of 1,233 patients with CKD who underwent flow cytometry-based assessment of T lymphocyte mitochondrial-related parameters during the study period were retrospectively screened. Of these, 67 patients were excluded for acute symptomatic infection, 52 for active immune disease, 78 for use of immunosuppressants, 226 for undergoing dialysis, and 8 for a history of kidney transplantation (Figure 1). Ultimately, a total of 802 patients were included in the analysis, comprising 440 males and 362 females, with a mean age of 56.3 ± 16.0 years.

The distribution of CKD stages among the patients was as follows: stage 1 (226), stage 2 (204), stage 3 (205), stage 4 (82), and stage 5 (85). General data are summarized in Table 1. Mean age was higher in CKD stages 3–4 (63.46 ± 13.65 years) and stage 5 (63.44 ± 14.47 years) compared with stages 1–2 (50.13 ± 15.14 years; *p* < 0.001). The prevalence of hypertension was higher in stages 3–4 (79.1%) and stage 5 (83.5%) than in stages 1–2 (51.4%; *p* < 0.001), as was diabetes (44.6 and 36.5% vs. 23.7%; *p* < 0.001) and cardiovascular disease (25.4 and 50.6% vs. 8.1%; *p* < 0.001). BMI was lower in stages 3–4 (23.89 ± 3.10 kg/m^2^) and stage 5 (23.73 ± 3.59 kg/m^2^) compared with stages 1–2 (24.77 ± 4.08 kg/m^2^; *p* = 0.009). A total of 467 patients underwent kidney biopsy with pathological confirmation.

**Table 1:** Demographics and clinical and laboratory parameters of patients at different stages of CKD.

Parameters	CKD 1-2 (*n* = 430)	CKD 3-4 (*n* = 287)	CKD 5 (*n* = 85)	*F*/χ^2^	*p*-Value
Gender, male [*n* (%)]	213 (49.5%)	182 (63.4%)	45 (52.9%)	13.532	0.001
Age (years)	50.13 ± 15.14	63.46 ± 13.65	63.44 ± 14.47	83.587	< 0.001
BMI (kg/m^2^)	24.77 ± 4.08	23.89 ± 3.10	23.73 ± 3.59	4.727	0.009
Hypertension [*n* (%)]	221 (51.4%)	227 (79.1%)	71 (83.5%)	72.570	< 0.001
Diabetes [*n* (%)]	102 (23.7%)	128 (44.6%)	31 (36.5%)	34.845	< 0.001
CVD history [*n* (%)]	35 (8.1%)	73 (25.4%)	43 (50.6%)	96.444	< 0.001
Cause of CKD					
IgA nephropathy	63 (26.25%)	33 (17.55%)	5 (12.82%)	65.387	< 0.001
Membranous nephropathy	60 (25.00%)	13 (6.91%)	0 (0%)		
Diabetic nephropathy	66 (27.50%)	109 (57.98%)	28 (71.80%)		
Other/unknown	51 (21.25%)	33 (17.55%)	6 (15.38%)		
Urine protein	291.79 (35.10, 1,159.07)	303.00 (35.58, 1,264.00)	596.72 (51.57, 2,061.25)	3.707	0.157
High-sensitivity C-reactive protein (mg/L)	0.50 (0.50, 0.61)	0.50 (0.50, 1.90)	1.05 (0.50, 7.86)	51.033	< 0.001
White blood cell count ( × 10^3^/mm^3^)	6.35 ± 1.75	6.34 ± 1.83	6.38 ± 1.70	0.014	0.986
Neutrophil percentage (%)	61.63 ± 9.22	64.62 ± 9.08	70.34 ± 9.58	33.895	< 0.001
Lymphocyte percentage (%)	28.55 ± 8.22	24.70 ± 7.92	18.09 ± 6.99	66.332	< 0.001
Hemoglobin (g/L)	135.33 ± 19.11	115.78 ± 19.91	88.11 ± 22.93	230.386	< 0.001
Platelet ( × 10^3^/mm^3^)	236.14 ± 64.87	210.70 ± 66.23	197.98 ± 75.45	19.057	< 0.001
Albumin (g/L)	38.02 ± 10.72	36.86 ± 6.03	33.95 ± 5.23	7.821	< 0.001
Creatinine (μmol/L)	75.87 ± 19.13	162.52 ± 58.15	593.12 ± 279.14	987.583	< 0.001
Uric acid (μmol/L)	349.93 ± 100.17	411.09 ± 124.03	441.35 ± 151.44	36.940	< 0.001
eGFR (ml/min/1.73 m^2^)	91.39 ± 19.77	38.80 ± 12.36	8.46 ± 3.38	1,444.015	< 0.001
Triglyceride (mmol/L)	1.85 ± 1.47	1.82 ± 1.07	1.64 ± 0.89	0.953	0.386
Total cholesterol (mmol/L)	5.43 ± 2.00	4.60 ± 1.75	4.30 ± 1.46	23.507	< 0.001
High-density lipoprotein cholesterol (mmol/L)	1.41 ± 0.51	1.21 ± 0.36	1.10 ± 0.32	27.710	< 0.001
Low-density lipoprotein cholesterol (mmol/L)	3.41 ± 1.37	2.86 ± 1.23	2.70 ± 1.04	20.638	< 0.001
Potassium (mmol/L)	3.98 ± 0.35	4.25 ± 0.54	4.56 ± 0.78	59.523	< 0.001
Calcium (mmol/L)	2.33 ± 0.09	2.33 ± 0.09	2.23 ± 0.17	31.767	< 0.001
Phosphorus (mmol/L)	1.14 ± 0.19	1.18 ± 0.21	1.72 ± 0.60	156.219	< 0.001

CKD, chronic kidney disease; BMI, body mass index; CVD, cardiovascular disease; eGFR, estimated glomerular filtration rate.

### Laboratory characteristics across CKD stages

Analysis of laboratory indicators across CKD stages 1–5 is presented in Table 1. While white blood cell count showed no significant differences (*p* = 0.986), neutrophil percentage was higher in more advanced CKD stages, with values of 61.63 ± 9.22% in CKD stages 1–2 and 70.34 ± 9.58% in CKD stage 5 (*p* < 0.001). Lymphocyte percentage was lower in CKD stage 5 compared with CKD stages 1–2 (28.55 ± 8.22% vs. 18.09 ± 6.99%, *p* < 0.001).

Hemoglobin declined from 135.33 ± 19.11 to 88.11 ± 22.93 g/L (*p* < 0.001), and platelet count from 236.14 ± 64.87 × 10^3^/m^3^ to 197.98 ± 75.45 × 10^3^/mm^3^ (*p* < 0.001). Serum creatinine rose sharply from 75.87 ± 19.13 μmol/L in CKD 1–2 to 593.12 ± 279.14 μmol/L in CKD 5 (*p* < 0.001), and uric acid increased from 349.93 ± 100.17 μmol/L to 441.35 ± 151.44 μmol/L (*p* < 0.001). Serum calcium fell from 2.33 ± 0.09 to 2.23 ± 0.17 mmol/L (*p* < 0.001), while total cholesterol, High-density lipoprotein cholesterol (HDL-C), and Low-density lipoprotein cholesterol (LDL-C) each showed significant decreasing trends (all *p* < 0.001). Potassium and phosphorus both increased significantly with advancing CKD stage (both *p* < 0.001). To further assess the potential impact of CKD etiology heterogeneity, subgroup multivariable regression analyses were performed after stratification by major CKD etiologies, including IgA nephropathy, membranous nephropathy, and diabetic nephropathy (Supplementary Table S1).

### Absolute counts of T-lymphocyte subsets

**Figure 2:**
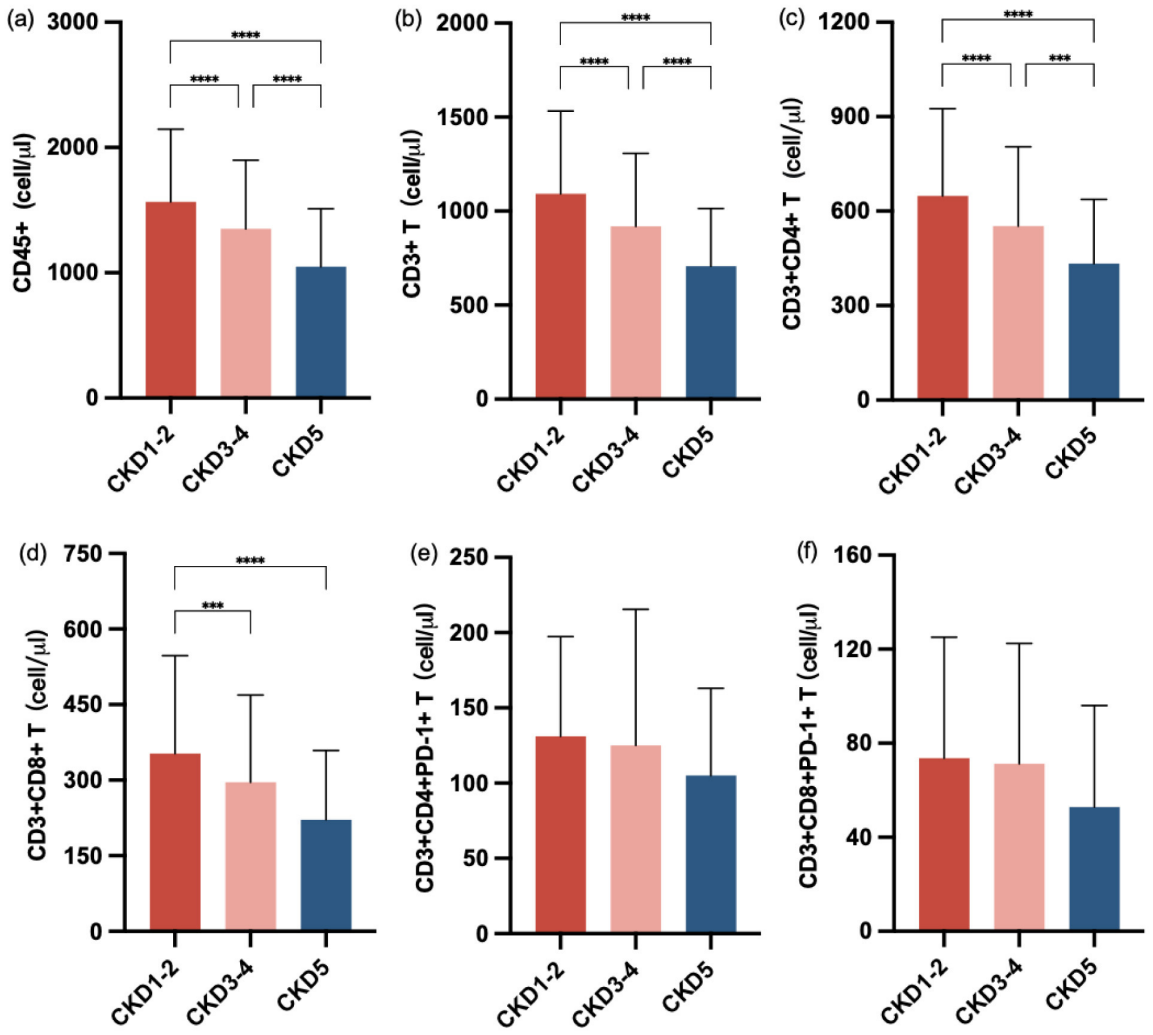
Absolute counts of T lymphocyte subsets in patients at different stages of chronic kidney disease. **(a)** CD45^+^ Lymphocyte absolute count. **(b)** CD3^+^ T Lymphocyte absolute count. **(c)** CD4^+^ T Lymphocyte absolute count. **(d)** CD8^+^ T Lymphocyte absolute count. **(e)** CD4^+^PD-1^+^ T Lymphocyte absolute count. **(f)** CD8^+^PD-1^+^ T Lymphocyte absolute count. CKD, chronic kidney disease. ***P < 0.001, ****P < 0.0001.

Comparative analysis of the absolute counts of T lymphocyte subsets revealed that the absolute counts of CD45^+^ lymphocytes and CD3^+^, CD4^+^, and CD8^+^ T lymphocytes were lower in more advanced CKD stages (Figure 2). In the Holm–Bonferroni sensitivity analysis, most pairwise differences in CD45^+^, CD3^+^, CD4^+^, and CD8^+^ T-cell absolute counts remained statistically significant, whereas the CKD stages 3–4 vs. stage 5 comparison for CD8^+^ T-cell absolute count and comparisons involving PD-1^+^ T-cell absolute counts were no longer significant after correction (Supplementary Table S2).

### Relative distribution of T-lymphocyte subsets

**Figure 3:**
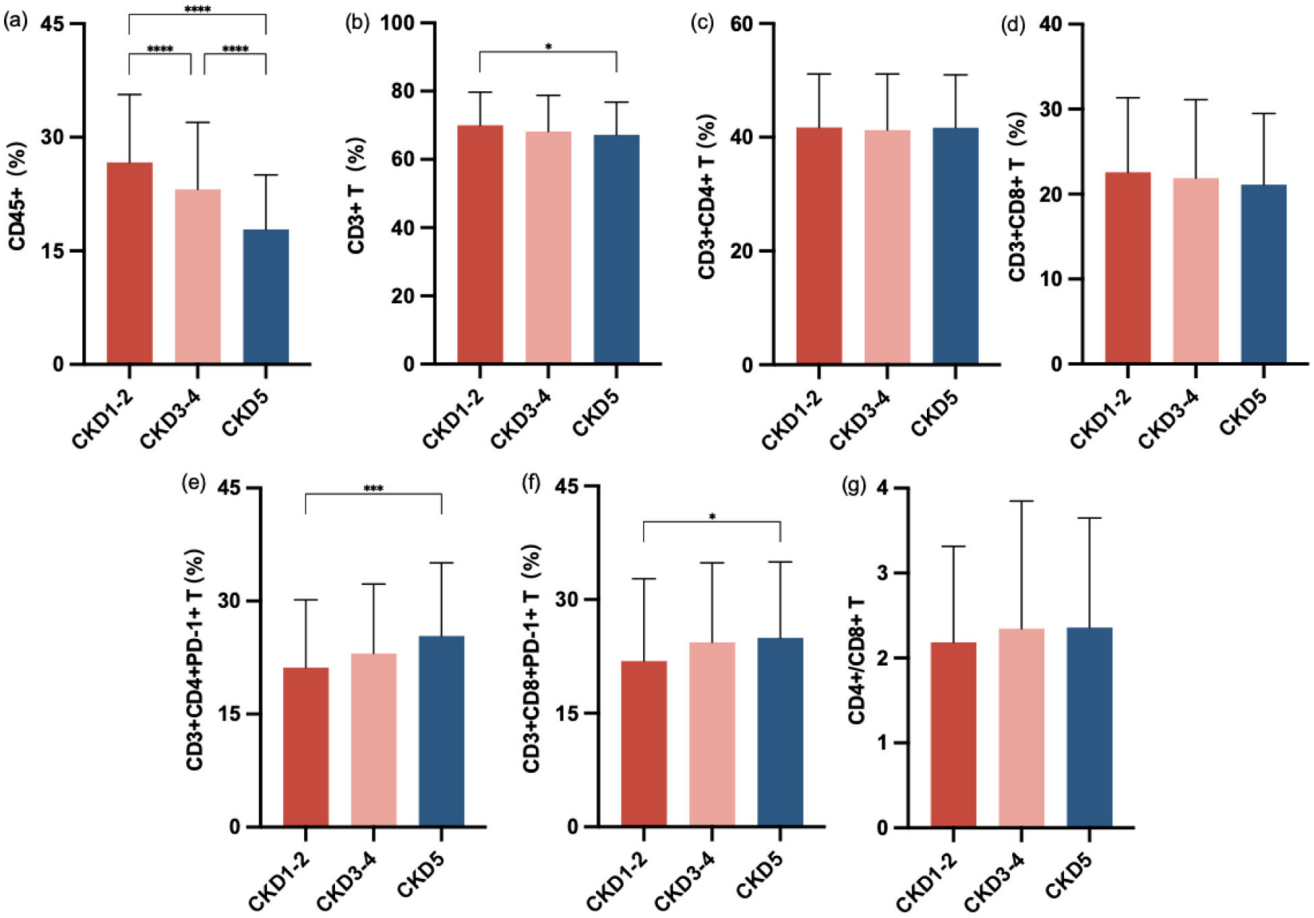
Percentages of T lymphocyte subsets in patients at different stages of chronic kidney disease. **(a)** CD45^+^ Lymphocyte percentage. **(b)** CD3^+^ T Lymphocyte percentage. **(c)** CD4^+^ T Lymphocyte percentage. **(d)** CD8^+^ T Lymphocyte percentage. **(e)** CD4^+^PD-1^+^ T Lymphocyte percentage. **(f)** CD8^+^PD-1^+^ T Lymphocyte percentage. **(g)** CD4^+^/CD8^+^ ratio. CKD, chronic kidney disease. *P < 0.05, ***P < 0.001, ****P < 0.0001.

Comparative analysis of the relative counts of T lymphocyte subsets showed that CD45^+^ lymphocyte percentage was lower in more advanced CKD stages (Figure 3). After Holm-Bonferroni correction, pairwise differences in CD45^+^ lymphocyte percentage remained statistically significant, whereas differences in CD3^+^, CD4^+^, and CD8^+^ T-cell percentages, CD4^+^/CD8^+^ ratio, and most PD-1^+^ T-cell percentage comparisons were not significant after correction, except for the CKD stages 1–2 vs. stage 5 comparison for CD4^+^PD-1^+^ T-cell percentage (Supplementary Table S2).

### Mitochondrial-related parameters of T lymphocytes across CKD stages

**Figure 4:**
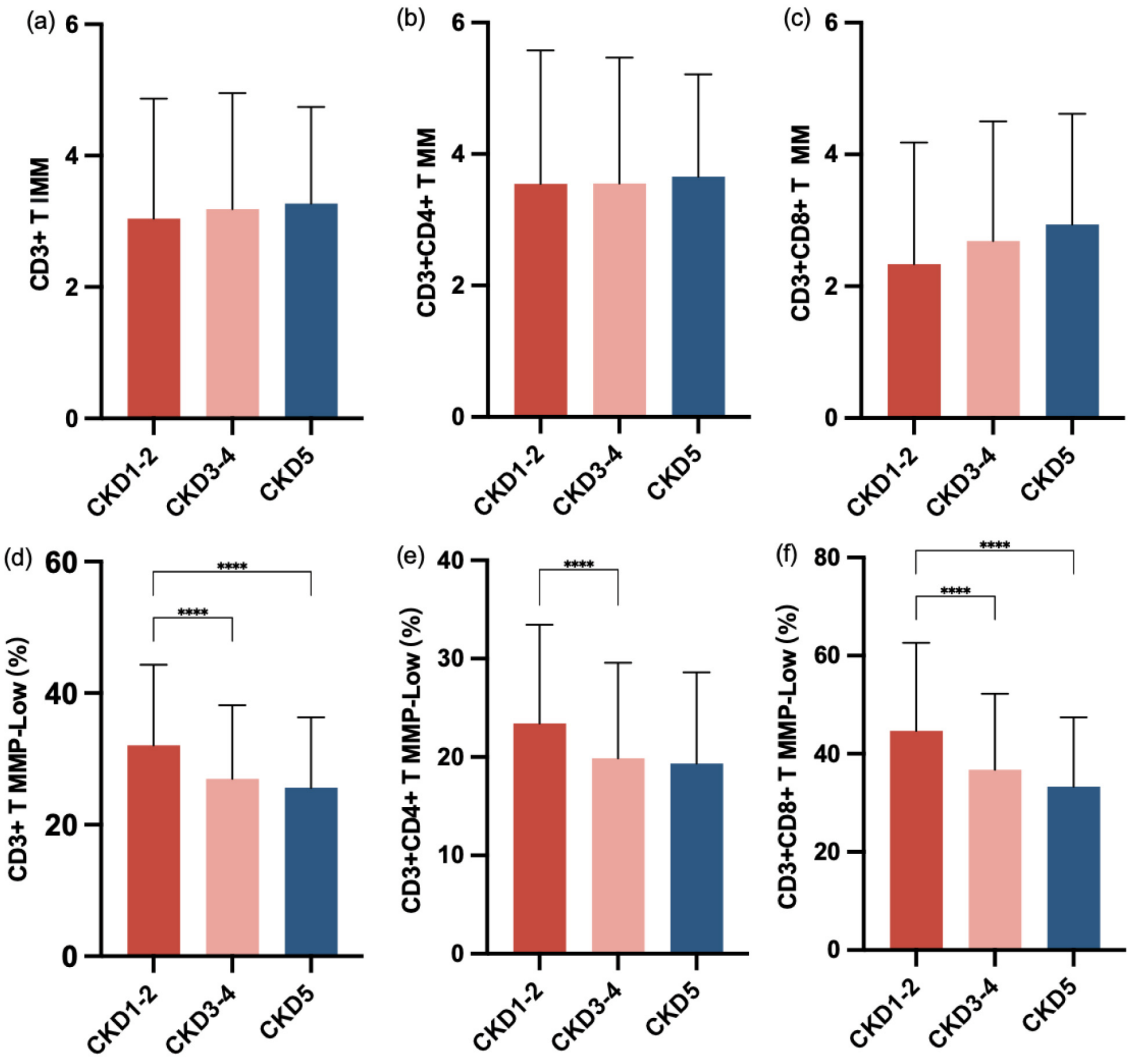
Mitochondrial-related parameters in patients at different stages of chronic kidney disease. **(a)** CD3^+^ T Lymphocyte MM. **(b)** CD4^+^ T Lymphocyte MM. **(c)** CD8^+^ T Lymphocyte MM. **(d)** CD3^+^ T Lymphocyte MMP-Low%. **(e)** CD4^+^ T Lymphocyte MMP-Low%. **(f)** CD8^+^ T Lymphocyte MMP-Low%. CKD, chronic kidney disease; MM, mitochondrial mass; MMP-Low%, percentage of cells with low mitochondrial membrane potential. ****P < 0.0001.

Analysis of MM and MMP-Low% is shown in Figure 4. MM of CD3^+^ and CD4^+^ T lymphocytes did not change significantly with CKD progression, whereas CD8^+^ T-cell MM differed across CKD stages before multiplicity correction. However, pairwise differences in CD8^+^ T-cell MM were no longer statistically significant after Holm-Bonferroni correction. The MMP-Low% of CD3^+^, CD4^+^, and CD8^+^ T lymphocytes was lower in more advanced CKD stages, with the main CKD stages 1–2 vs. stages 3–4 comparisons for all three T-cell subsets and the CKD stages 1–2 vs. stage 5 comparisons for CD3^+^ and CD8^+^ T-cell MMP-Low% remaining significant after correction (Supplementary Table S2).

### Association between eGFR and T-lymphocyte mitochondrial-related parameters

Bivariate correlation analysis showed that eGFR was positively correlated with the absolute counts of CD45^+^ lymphocytes (*r* = 0.301, P-FDR < 0.001), CD3^+^ T cells (*r* = 0.328, P-FDR < 0.001), CD4^+^ T cells (*r* = 0.291, P-FDR < 0.001), and CD8^+^ T cells (*r* = 0.286, P-FDR < 0.001), as well as with the percentage of CD45^+^ lymphocytes (*r* = 0.311, P-FDR < 0.001). eGFR was also positively correlated with the MMP-Low% of CD3^+^ (*r* = 0.268, P-FDR < 0.001), CD4^+^ (*r* = 0.212, P-FDR < 0.001), and CD8^+^ T lymphocytes (*r* = 0.307, P-FDR < 0.001). These associations remained significant after both FDR and Bonferroni correction (Table 2).

**Table 2:** Bivariate correlation between eGFR and T lymphocyte subsets and mitochondrial-related parameters (*n* = 802).

Parameters	*r*	*p*-Value	P-FDR	P-Bonferroni
CD45^+^ absolute count (cell/μl)	0.301	< 0.001	< 0.001	< 0.001
CD3^+^ T absolute count (cell/μl)	0.328	< 0.001	< 0.001	< 0.001
CD4^+^ T absolute count (cell/μl)	0.291	< 0.001	< 0.001	< 0.001
CD8^+^T absolute count (cell/μl)	0.286	< 0.001	< 0.001	< 0.001
CD4^+^PD-1^+^ T absolute count (cell/μl)	0.118	0.001	0.001	0.015
CD8^+^PD-1^+^ T absolute count (cell/μl)	0.139	< 0.001	< 0.001	0.002
CD45^+^ percentage (%)	0.311	< 0.001	< 0.001	< 0.001
CD3^+^T percentage (%)	0.110	0.002	0.003	0.035
CD4^+^T percentage (%)	0.016	0.649	0.649	1.000
CD8^+^T percentage (%)	0.094	0.008	0.009	0.151
CD4^+^PD-1^+^ T percentage (%)	−0.191	< 0.001	< 0.001	< 0.001
CD8^+^PD-1^+^ T percentage (%)	−0.158	< 0.001	< 0.001	< 0.001
CD4^+^/CD8^+^ ratio	−0.062	0.080	0.089	1.000
CD3^+^ T MM	−0.110	0.002	0.003	0.034
CD4^+^ T MM	−0.050	0.156	0.164	1.000
CD8^+^ T MM	−0.208	< 0.001	< 0.001	< 0.001
CD3^+^ T MMP-Low%	0.268	< 0.001	< 0.001	< 0.001
CD4^+^ T MMP-Low%	0.212	< 0.001	< 0.001	< 0.001
CD8^+^ T MMP-Low%	0.307	< 0.001	< 0.001	< 0.001

eGFR, estimated glomerular filtration rate; MM, mitochondrial mass; MMP-Low%, percentage of cells with low mitochondrial membrane potential; FDR, false discovery rate. Correlations were assessed using Spearman correlation analysis. P-FDR values were adjusted using the Benjamini–Hochberg method. P-Bonferroni values were adjusted using Bonferroni correction.

**Figure 5:**
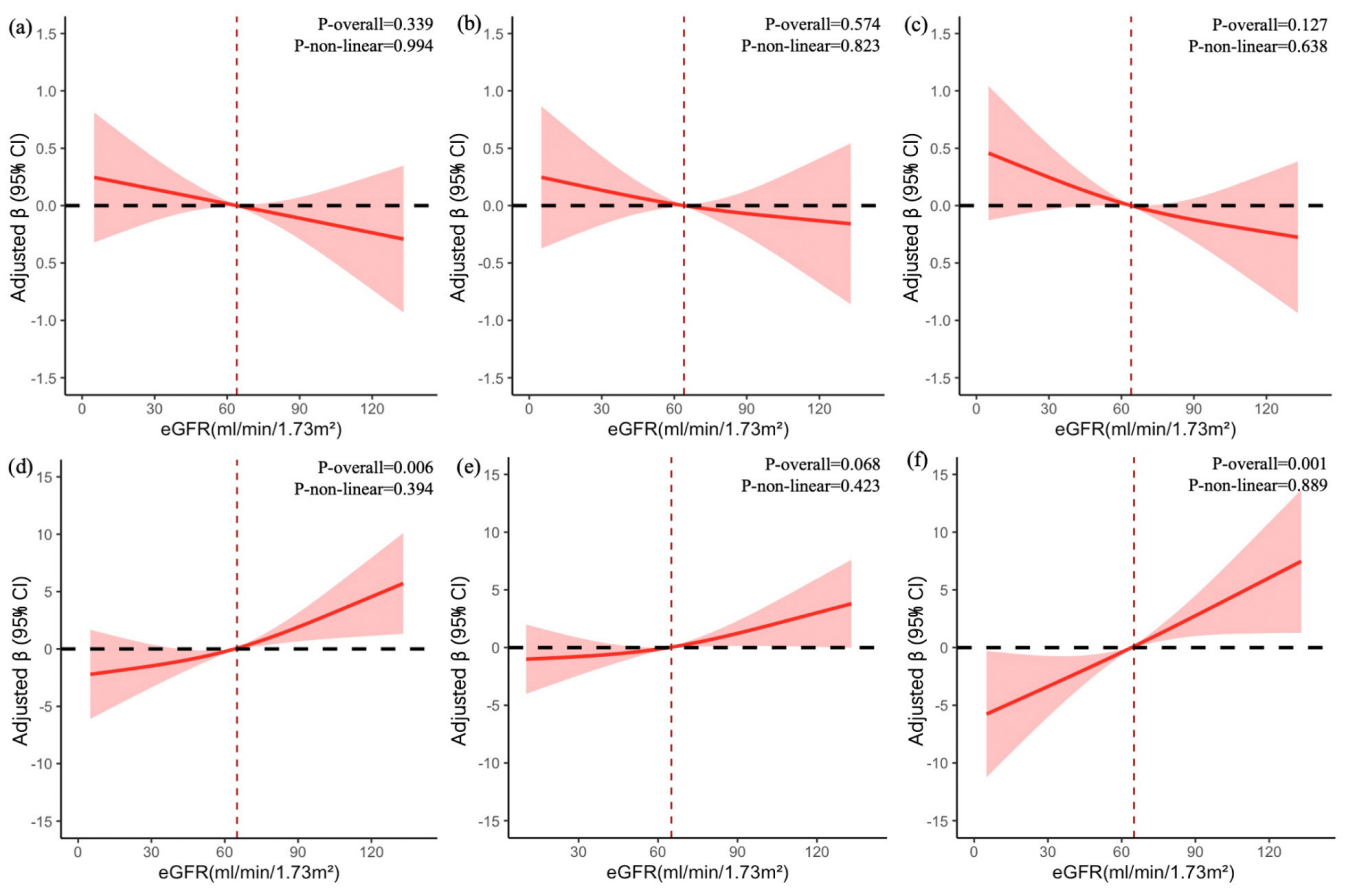
Restricted cubic spline regression analysis of eGFR with MM and MMP-Low% of T lymphocytes adjusted for gender, age, diabetes, hypertension, CVD, BMI, cause of CKD, high-sensitivity C-reactive protein, white blood cell count, hemoglobin, albumin, and total cholesterol. **(a)** MM of CD3^+^ T lymphocytes; **(b)** MM of CD4^+^ T lymphocytes; **(c)** MM of CD8^+^ T lymphocytes; **(d)** MMP-Low% of CD3^+^ T lymphocytes; **(e)** MMP-Low% of CD4^+^ T lymphocytes; **(f)** MMP-Low% of CD8^+^ T lymphocytes. eGFR, estimated glomerular filtration rate; MM, mitochondrial mass; MMP-Low%, percentage of cells with low mitochondrial membrane potential; BMI, body mass index; CVD, cardiovascular disease.

Linear regression analysis with mitochondrial-related parameters of T lymphocytes as dependent variables indicated that eGFR was significantly associated with the MM of CD3^+^ and CD8^+^ T lymphocytes and the MMP-Low% of CD3^+^, CD4^+^, and CD8^+^ T lymphocytes in univariate models. After adjusting for gender, age, diabetes mellitus, hypertension, Cardiovascular disease (CVD), BMI, cause of CKD, high-sensitivity C-reactive protein, white blood cell count, hemoglobin, albumin, and total cholesterol, eGFR remained independently associated with the MMP-Low% of CD3^+^ T lymphocytes (β = 0.0375, *p* = 0.024) and CD8^+^ T lymphocytes (β = 0.0826, *p* < 0.001), whereas the associations with CD3^+^, CD4^+^, and CD8^+^ T-cell MM and CD4^+^ T-cell MMP-Low% were no longer statistically significant (Table 3). Robust analyses using heteroscedasticity-consistent standard errors did not materially change these conclusions. Model diagnostics showed no substantial multicollinearity, with all maximum VIF values below 3. The restricted cubic spline analysis showed no evidence of nonlinearity in the associations between eGFR and MM or MMP-Low% of T lymphocytes (*p* for nonlinearity > 0.05; Figure 5).

**Table 3:** Linear regression analysis of eGFR and mitochondrial-related parameters of T lymphocytes.

Parameter	Univariate β (95% CI)	Univariate *p*-Value	Multivariable β (95% CI)	Multivariable *p*-Value	Robust β (95% CI)	Robust *p*-Value
CD3^+^ T MM	−0.0039 (−0.0074, −0.0004)	0.031	0.0004 (−0.0038, 0.0046)	0.840	0.0004 (−0.0036, 0.0045)	0.834
CD4^+^ T MM	−0.0014 (−0.0053, 0.0024)	0.471	0.0003 (−0.0042, 0.0048)	0.901	0.0003 (−0.0040, 0.0046)	0.897
CD8^+^ T MM	−0.0084 (−0.0120, −0.0048)	< 0.001	−0.0019 (−0.0063, 0.0026)	0.404	−0.0019 (−0.0062, 0.0024)	0.389
CD3^+^ T MMP-Low%	0.0952 (0.0723, 0.1182)	< 0.001	0.0375 (0.0051, 0.0700)	0.024	0.0375 (0.0044, 0.0707)	0.027
CD4^+^ T MMP-Low%	0.0602 (0.0408, 0.0796)	< 0.001	0.0211 (−0.0070, 0.0492)	0.140	0.0211 (−0.0085, 0.0507)	0.163
CD8^+^ T MMP-Low%	0.1579 (0.1254, 0.1903)	< 0.001	0.0826 (0.0367, 0.1285)	< 0.001	0.0826 (0.0371, 0.1281)	< 0.001

Model 1: univariate linear regression analysis without adjustment. Model 2: multivariable linear regression analysis adjusted for selected covariates. Robust estimates were calculated using heteroscedasticity-consistent standard errors.

## Discussion

This study demonstrated that more advanced CKD stages were associated with lower absolute counts of CD3^+^, CD4^+^, and CD8^+^ T lymphocytes and altered mitochondrial-related parameters, characterized by increased mitochondrial mass in CD8^+^ T cells and reduced MMP-Low% across T-cell subsets. After multivariable adjustment, eGFR remained independently associated with CD3^+^ and CD8^+^ T-cell MMP-Low%, whereas the association with CD8^+^ T-cell mitochondrial mass was no longer statistically significant. Clinically, these findings suggest that immune cell depletion and mitochondrial-related alterations may reflect immune dysregulation accompanying CKD progression and could serve as complementary indicators of disease severity and systemic inflammatory status.

From a clinical perspective, these immunometabolic alterations may have potential translational relevance. The consistent associations between eGFR, T lymphocyte counts, and mitochondrial-related parameters suggest that these features may represent candidate biomarkers of CKD severity and systemic immune dysregulation. In particular, mitochondrial-related parameters in CD8^+^ T lymphocytes may provide additional insight into immune-metabolic alterations associated with renal dysfunction. However, given the cross-sectional design of the present study, it remains unclear whether these alterations have predictive value for disease progression or clinical outcomes. Future longitudinal studies incorporating prognostic analyses are required to determine their potential utility in risk stratification and disease monitoring.

Previous studies have confirmed that immune dysregulation is a prominent feature of CKD, with multiple immune cell populations and cytokines involved in disease development and progression (41). In ESRD, reduced percentages of CD3^+^ T lymphocytes, decreased absolute counts of CD4^+^ and CD8^+^ T lymphocytes, and a lower CD4^+^/CD8^+^ ratio have been reported (42, 43), although inconsistent associations between T lymphocyte subsets and CKD stage have also been described (20). In addition, lower absolute counts of T lymphocyte subsets in CKD stages 3–5 compared with healthy controls have been observed 6. Consistent with these findings, the present study showed that absolute counts of CD3^+^, CD4^+^, and CD8^+^ T lymphocytes were lower in CKD stages 3–5 than in stages 1–2, whereas relative proportions of CD4^+^ and CD8^+^ T lymphocytes and the CD4^+^/CD8^+^ ratio were largely preserved. Correlation analysis further indicated that absolute T lymphocyte counts were more strongly associated with eGFR than relative proportions. Together, these findings suggest that quantitative T-cell depletion, rather than subset imbalance, characterizes immune alterations in advanced CKD, consistent with uremia-associated immune aging and loss of naïve T cells (44). Reduced levels of CD3^+^, CD4^+^, and CD8^+^ T lymphocytes have also been linked to adverse renal outcomes and infections (19).

Importantly, MM and MMP-Low% are flow cytometry–based parameters that reflect mitochondrial content and membrane potential distribution, respectively, and serve as indirect surrogate markers rather than direct measures of mitochondrial respiratory function, ATP production, reactive oxygen species generation, or mitochondrial dynamics. The mitochondrial alterations observed in this study, namely increased MM in CD8^+^ T lymphocytes together with a lower percentage of cells with MMP-Low%, should not be interpreted as evidence of enhanced mitochondrial or immune cell function. Such an interpretation would be inconsistent with the established pathophysiology of advanced CKD. Instead, these findings are more consistent with mitochondrial dysfunction and stress-related compensatory responses in T lymphocytes. A lower MMP-Low% may reflect a shift toward mitochondrial membrane hyperpolarization, a state that has been associated with mitochondrial stress, increased reactive oxygen species production, and heightened susceptibility to apoptosis in chronically stimulated immune cells (45). In this context, a reduced proportion of cells with low membrane potential does not necessarily indicate improved mitochondrial fitness, but may instead represent maladaptive bioenergetic remodeling associated with cellular stress. However, the interpretation of decreased MMP-Low% as reflecting mitochondrial hyperpolarization or stress-related responses remains inferential and lacks direct functional validation in the present study.

Similarly, the increase in MM observed in CD8^+^ T lymphocytes may reflect a compensatory response to mitochondrial injury rather than enhanced bioenergetic capacity. Experimental models have shown that mitochondrial dysfunction in T cells can induce compensatory mitochondrial biogenesis or accumulation of structurally abnormal mitochondria, yet remain associated with impaired respiratory function, increased inflammatory signaling, and features of premature immune senescence (46). Such compensatory increases in mitochondrial content may therefore coexist with, rather than correct, underlying mitochondrial dysfunction.

Taken together, these findings suggest that the mitochondrial alterations observed in T lymphocytes in advanced CKD reflect dysfunctional remodeling and stress compensation, rather than improved mitochondrial performance, consistent with chronic inflammatory and metabolic stress characteristic of advanced renal disease.

Persistent inflammation in CKD is associated with increased oxidative stress, which may contribute to T-cell activation and exhaustion. PD-1 is an immune checkpoint receptor predominantly expressed on T lymphocytes, and engagement with its ligands PD-L1 or PD-L2 suppresses T-cell proliferation, activation, cytokine production, metabolic activity, and cytotoxic function, thereby promoting T-cell exhaustion or apoptosis (47, 48). In the present study, absolute counts of CD4^+^PD-1^+^ and CD8^+^PD-1^+^ T lymphocytes decreased with CKD progression, whereas their relative proportions increased, suggesting differential vulnerability of PD-1-expressing T-cell subsets in advanced CKD. This pattern was more evident in advanced CKD stages and is consistent with the findings reported by Hartzell et al. (49).

Consistent with the above findings, CD8^+^ T lymphocytes in advanced CKD exhibited higher MM and lower MMP-Low%, further supporting the presence of altered mitochondrial-related parameters in more advanced CKD. Additionally, MMP-Low% levels of CD3^+^ and CD4^+^ T lymphocytes in CKD stages 3–5 were also lower compared to CKD stages 1–2, indicating alterations in mitochondrial parameters in peripheral blood T lymphocytes, particularly CD8^+^ T cells, which were associated with lower eGFR levels. The pathophysiological mechanisms underlying the mitochondrial changes in T lymphocytes during the progression of CKD may be attributed to uremic toxins and the inflammatory state. Previous research has shown that HK2 cells treated with uremic toxins, namely indoxyl sulfate (IS) and p-cresol sulfate (PCS), exhibited a higher MMP compared to control cells. Moreover, *in vitro* studies have demonstrated that IS and PCS can reduce MM through the autophagic machinery (50, 51). In muscle cells, IS has been found to induce the disintegration of the mitochondrial network and an increase in mitochondrial fission (52). However, the relationship between uremic toxins and the MM and MMP of T lymphocytes has not been reported. In our study, although MMP-low% decreased as renal function progressed, the MM of CD8^+^ T lymphocytes increased, which is inconsistent with previous findings. This suggests the existence of different molecular mechanisms that warrant further investigation. Additionally, circulating inflammatory mediators may exacerbate mitochondrial dysfunction through several upstream molecular pathways. For instance, elevated IL-6 in myotubes leads to impaired mitochondrial capacity and disruption of muscle metabolic homeostasis (53). In liver biopsies of patients with type 2 diabetes mellitus (T2DM), mitochondrial dynamics decline and tend to fuse under inflammatory conditions (54). Another study revealed that the MMP-low% of CD8^+^ T cells was significantly decreased in liver cirrhosis patients and a correlation was confirmed between MMP-low% and MM, early liver inflammation, and the progression of hepatitis B virus (HBV) infection (32). Thus, we hypothesized that alterations in MM and MMP-Low% of T lymphocytes are implicated in CKD-related inflammation and warrant further investigation.

Previous studies indicated that aging could lead to immune senescence, with decreases in T lymphocytes (55) and MM (56). In this study, although patients in advanced CKD stages were older, MM of CD8^+^ T lymphocytes was higher rather than lower. In addition, after adjusting for multiple factors including age in the linear regression analysis, eGFR remained associated with MMP-Low% of CD3^+^ and CD8^+^ T lymphocytes, suggesting that these associations were independent of aging. It was reported that HIV infection could lead to an increase in MM in CD4^+^ T and CD8^+^ T cells, but mainly influenced MMP in CD8^+^ T cells (57). In contrast, this study revealed that CKD progression led to an increase in MM in CD8^+^ T cells, while MMP-low% of CD4^+^ T cells and CD8^+^ T cells decreased. The results showed that the influence of CKD progression on MMP was more obvious. However, further studies are needed to determine the underlying mechanisms linking CKD stage and mitochondrial alterations.

This study has several limitations. First, only a portion of the patients underwent kidney biopsy for pathological classification, which may have limited the ability to account for disease heterogeneity. Second, although patients receiving immunosuppressive therapy were excluded, the effects of medications commonly used in patients with CKD, such as renin-angiotensin system inhibitors (ACE inhibitors or angiotensin receptor blockers), statins, metformin, and other metabolic or cardiovascular agents, on T lymphocyte subsets and mitochondrial-related parameters were not adjusted for, which represents an important source of potential residual confounding. In addition, although subgroup analyses stratified by major CKD etiologies showed similar trends, residual confounding related to disease heterogeneity cannot be fully excluded. Third, no longitudinal follow-up was performed, precluding within-individual comparisons over time. Fourth, owing to the cross-sectional design, the observed associations between renal function, T lymphocyte alterations, and mitochondrial parameters should be interpreted as correlational rather than causal. It remains unclear whether mitochondrial alterations represent causes, consequences, or parallel phenomena of CKD progression, and prospective longitudinal studies are required to clarify temporal and mechanistic relationships. In addition, the absence of a healthy control group limits the ability to determine whether the observed immunological and mitochondrial alterations are already present in early-stage CKD or represent progressive deviations from normal physiological states, and future studies incorporating healthy controls are warranted. Finally, specific molecular mechanisms were not investigated, and further mechanistic studies are warranted.

## Conclusions

In patients with CKD, there is a notable decrease in T lymphocyte numbers and alterations in mitochondrial-related parameters. Alterations in T lymphocyte counts and mitochondrial-related parameters were associated with more advanced CKD stages. These findings may represent CKD severity-associated immunometabolic phenotypes and provide insights into immune dysregulation in CKD. However, their potential clinical utility for disease monitoring or prognostic assessment requires further validation in longitudinal studies.

## Data Availability

The original contributions presented in the study are included in the article/Supplementary material, further inquiries can be directed to the corresponding author.
