# Supplementary material for: Alterations in T lymphocyte subsets and mitochondrial-related parameters in patients with chronic kidney disease
Source: Front Med (Lausanne). 2026 Jun 19;13:1811069. doi: 10.3389/fmed.2026.1811069 (PMC13327998; doi:10.3389/fmed.2026.1811069)
Supplement: Supplementary file 1 [file Table_1.docx]

**Supplementary Table S1. Subgroup multivariable regression analyses stratified by CKD etiology**

| **CKD etiology** | **n** | **Outcome** | **eGFR β** | **SE** | **95% CI** | **P value** | **FDR-adjusted P** | **R²** |
| --- | --- | --- | --- | --- | --- | --- | --- | --- |
| IgA nephropathy | 110 | CD3⁺ T MM | -0.098 | 0.284 | -0.664 to 0.467 | 0.731 | 0.869 | 0.032 |
| IgA nephropathy | 110 | CD4⁺ T MM | -0.052 | 0.314 | -0.672 to 0.568 | 0.869 | 0.869 | 0.015 |
| IgA nephropathy | 110 | CD8⁺ T MM | -0.286 | 0.312 | -0.906 to 0.335 | 0.364 | 0.739 | 0.054 |
| IgA nephropathy | 110 | CD3⁺ T MMP-Low% | -0.992 | 1.440 | -3.852 to 1.867 | 0.493 | 0.739 | 0.075 |
| IgA nephropathy | 110 | CD4⁺ T MMP-Low% | -1.964 | 1.192 | -4.333 to 0.405 | 0.103 | 0.619 | 0.101 |
| IgA nephropathy | 110 | CD8⁺ T MMP-Low% | 1.642 | 2.153 | -2.638 to 5.922 | 0.449 | 0.739 | 0.069 |
| Membranous nephropathy | 73 | CD3⁺ T MM | 0.201 | 0.303 | -0.404 to 0.806 | 0.509 | 0.611 | 0.082 |
| Membranous nephropathy | 73 | CD4⁺ T MM | 0.269 | 0.335 | -0.398 to 0.935 | 0.424 | 0.611 | 0.071 |
| Membranous nephropathy | 73 | CD8⁺ T MM | 0.029 | 0.289 | -0.547 to 0.605 | 0.920 | 0.920 | 0.094 |
| Membranous nephropathy | 73 | CD3⁺ T MMP-Low% | 6.405 | 2.266 | 1.894 to 10.916 | 0.006 | 0.018 | 0.288 |
| Membranous nephropathy | 73 | CD4⁺ T MMP-Low% | 5.617 | 2.077 | 1.463 to 9.770 | 0.009 | 0.018 | 0.275 |
| Membranous nephropathy | 73 | CD8⁺ T MMP-Low% | 8.289 | 2.986 | 2.349 to 14.230 | 0.007 | 0.018 | 0.300 |
| Diabetic nephropathy | 203 | CD3⁺ T MM | -0.246 | 0.146 | -0.534 to 0.042 | 0.094 | 0.113 | 0.036 |
| Diabetic nephropathy | 203 | CD4⁺ T MM | -0.237 | 0.162 | -0.556 to 0.082 | 0.145 | 0.145 | 0.030 |
| Diabetic nephropathy | 203 | CD8⁺ T MM | -0.363 | 0.150 | -0.658 to -0.067 | 0.017 | 0.050 | 0.062 |
| Diabetic nephropathy | 203 | CD3⁺ T MMP-Low% | 1.632 | 0.970 | -0.276 to 3.540 | 0.093 | 0.113 | 0.035 |
| Diabetic nephropathy | 203 | CD4⁺ T MMP-Low% | 1.458 | 0.867 | -0.243 to 3.159 | 0.092 | 0.113 | 0.030 |
| Diabetic nephropathy | 203 | CD8⁺ T MMP-Low% | 3.623 | 1.309 | 1.050 to 6.195 | 0.006 | 0.036 | 0.066 |

**Note:** CKD, chronic kidney disease; eGFR, estimated glomerular filtration rate; MM, mitochondrial mass; MMP-Low%, percentage of cells with low mitochondrial membrane potential; SE, standard error; CI, confidence interval; FDR, false discovery rate. Subgroup analyses were performed using multivariable linear regression models stratified by CKD etiology. eGFR and age were standardized as z-scores, and coefficients represent the change in outcome per 1-SD increase in eGFR. Models included eGFR, sex, age, diabetes, hypertension, and high-sensitivity C-reactive protein. P values were adjusted using the Benjamini–Hochberg FDR method. Collinearity assessment showed no substantial multicollinearity, with all variance inflation factors <5.

**Supplementary Table S2. Sensitivity analysis of Holm-Bonferroni-corrected pairwise comparisons across CKD stages**

| **Parameter** | **Comparison** | **Raw P** | **Holm-Bonferroni-adjusted P** |
| --- | --- | --- | --- |
| CD45+ lymphocyte absolute count | CKD 1–2 vs CKD 3–4 | <0.001 | <0.001 |
| CD45+ lymphocyte absolute count | CKD 1–2 vs CKD 5 | <0.001 | <0.001 |
| CD45+ lymphocyte absolute count | CKD 3–4 vs CKD 5 | <0.001 | 0.002 |
| CD3+ T absolute count | CKD 1–2 vs CKD 3–4 | <0.001 | <0.001 |
| CD3+ T absolute count | CKD 1–2 vs CKD 5 | <0.001 | <0.001 |
| CD3+ T absolute count | CKD 3–4 vs CKD 5 | <0.001 | 0.004 |
| CD4+ T absolute count | CKD 1–2 vs CKD 3–4 | <0.001 | <0.001 |
| CD4+ T absolute count | CKD 1–2 vs CKD 5 | <0.001 | <0.001 |
| CD4+ T absolute count | CKD 3–4 vs CKD 5 | <0.001 | 0.025 |
| CD8+ T absolute count | CKD 1–2 vs CKD 3–4 | <0.001 | 0.006 |
| CD8+ T absolute count | CKD 1–2 vs CKD 5 | <0.001 | <0.001 |
| CD8+ T absolute count | CKD 3–4 vs CKD 5 | 0.003 | 0.090 |
| CD4+PD-1+ T absolute count | CKD 1–2 vs CKD 3–4 | 0.548 | 1.000 |
| CD4+PD-1+ T absolute count | CKD 1–2 vs CKD 5 | 0.010 | 0.316 |
| CD4+PD-1+ T absolute count | CKD 3–4 vs CKD 5 | 0.078 | 1.000 |
| CD8+PD-1+ T absolute count | CKD 1–2 vs CKD 3–4 | 0.799 | 1.000 |
| CD8+PD-1+ T absolute count | CKD 1–2 vs CKD 5 | 0.001 | 0.055 |
| CD8+PD-1+ T absolute count | CKD 3–4 vs CKD 5 | 0.009 | 0.294 |
| CD45+ lymphocyte percentage | CKD 1–2 vs CKD 3–4 | <0.001 | <0.001 |
| CD45+ lymphocyte percentage | CKD 1–2 vs CKD 5 | <0.001 | <0.001 |
| CD45+ lymphocyte percentage | CKD 3–4 vs CKD 5 | <0.001 | <0.001 |
| CD3+ T percentage | CKD 1–2 vs CKD 3–4 | 0.047 | 1.000 |
| CD3+ T percentage | CKD 1–2 vs CKD 5 | 0.136 | 1.000 |
| CD3+ T percentage | CKD 3–4 vs CKD 5 | 0.926 | 1.000 |
| CD4+ T percentage | CKD 1–2 vs CKD 3–4 | 0.812 | 1.000 |
| CD4+ T percentage | CKD 1–2 vs CKD 5 | 0.997 | 1.000 |
| CD4+ T percentage | CKD 3–4 vs CKD 5 | 0.950 | 1.000 |
| CD8+ T percentage | CKD 1–2 vs CKD 3–4 | 0.566 | 1.000 |
| CD8+ T percentage | CKD 1–2 vs CKD 5 | 0.352 | 1.000 |
| CD8+ T percentage | CKD 3–4 vs CKD 5 | 0.764 | 1.000 |
| CD4+PD-1+ T percentage | CKD 1–2 vs CKD 3–4 | 0.021 | 0.627 |
| CD4+PD-1+ T percentage | CKD 1–2 vs CKD 5 | <0.001 | 0.013 |
| CD4+PD-1+ T percentage | CKD 3–4 vs CKD 5 | 0.096 | 1.000 |
| CD8+PD-1+ T percentage | CKD 1–2 vs CKD 3–4 | 0.007 | 0.237 |
| CD8+PD-1+ T percentage | CKD 1–2 vs CKD 5 | 0.180 | 1.000 |
| CD8+PD-1+ T percentage | CKD 3–4 vs CKD 5 | 0.984 | 1.000 |
| CD4+/CD8+ ratio | CKD 1–2 vs CKD 3–4 | 0.232 | 1.000 |
| CD4+/CD8+ ratio | CKD 1–2 vs CKD 5 | 0.485 | 1.000 |
| CD4+/CD8+ ratio | CKD 3–4 vs CKD 5 | 0.995 | 1.000 |
| CD3+ T MM | CKD 1–2 vs CKD 3–4 | 0.535 | 1.000 |
| CD3+ T MM | CKD 1–2 vs CKD 5 | 0.511 | 1.000 |
| CD3+ T MM | CKD 3–4 vs CKD 5 | 0.914 | 1.000 |
| CD4+ T MM | CKD 1–2 vs CKD 3–4 | 1.000 | 1.000 |
| CD4+ T MM | CKD 1–2 vs CKD 5 | 0.891 | 1.000 |
| CD4+ T MM | CKD 3–4 vs CKD 5 | 0.903 | 1.000 |
| CD8+ T MM | CKD 1–2 vs CKD 3–4 | 0.031 | 0.886 |
| CD8+ T MM | CKD 1–2 vs CKD 5 | 0.015 | 0.471 |
| CD8+ T MM | CKD 3–4 vs CKD 5 | 0.508 | 1.000 |
| CD3+ T MMP-Low% | CKD 1–2 vs CKD 3–4 | <0.001 | <0.001 |
| CD3+ T MMP-Low% | CKD 1–2 vs CKD 5 | <0.001 | <0.001 |
| CD3+ T MMP-Low% | CKD 3–4 vs CKD 5 | 0.634 | 1.000 |
| CD4+ T MMP-Low% | CKD 1–2 vs CKD 3–4 | <0.001 | <0.001 |
| CD4+ T MMP-Low% | CKD 1–2 vs CKD 5 | 0.002 | 0.055 |
| CD4+ T MMP-Low% | CKD 3–4 vs CKD 5 | 0.902 | 1.000 |
| CD8+ T MMP-Low% | CKD 1–2 vs CKD 3–4 | <0.001 | <0.001 |
| CD8+ T MMP-Low% | CKD 1–2 vs CKD 5 | <0.001 | <0.001 |
| CD8+ T MMP-Low% | CKD 3–4 vs CKD 5 | 0.218 | 1.000 |

**Note:** CKD, chronic kidney disease; MM, mitochondrial mass; MMP-Low%, percentage of cells with low mitochondrial membrane potential. Pairwise comparisons among CKD stages were initially performed using Tukey’s HSD test. Holm-Bonferroni correction was applied across all 57 pairwise comparisons involving 19 immune and mitochondrial parameters. A Holm-Bonferroni-adjusted P value <0.05 was considered statistically significant
